# Circadian-Tuned Peptide Drug/Gene Co-Delivery Nanocomplexes to Enhance Glioblastoma Targeting and Transfection

**DOI:** 10.3390/ijms26136130

**Published:** 2025-06-26

**Authors:** Ana R. Neves, Eric Vivès, Prisca Boisguérin, Telma Quintela, Diana Costa

**Affiliations:** 1 RISE-Health, Department of Medical Sciences, Faculty of Health Sciences, University of Beira Interior, Av. Infante D. Henrique, 6200-506 Covilhã, Portugal; ana.neves@ubi.pt (A.R.N.); tquintela@fcsaude.ubi.pt (T.Q.); 2 PhyMedExp, University of Montpellier, INSERM, CNRS, 34295 Montpellier, France; eric.vives@umontpellier.fr (E.V.); prisca.boisguerin@inserm.fr (P.B.); 3 BRIDGES-Biotechnology Research, Innovation and Design for Health Products, Polytechnic University of Guarda, 6300-559 Guarda, Portugal

**Keywords:** chronotherapy, circadian rhythms, drug/gene dual therapy, glioblastoma, nanotechnology, TMZ/*TP53* systems

## Abstract

Glioblastoma is the most prevalent and aggressive form of brain malignancy. Actual treatments face several challenges due to its high aggressiveness and poor prognosis. The chemotherapeutic agent temozolomide (TMZ) has limited therapeutic efficacy, and mutations in the tumour protein p53 gene (*TP53*) have been associated with treatment resistance. Thus, this study aimed to explore an innovative therapeutic strategy to enhance treatment efficacy of GBM. Previously, our team had developed a WRAP5 cell-penetrating peptide (CPP) functionalized with a transferrin receptor ligand (Tf) for the targeted delivery of TMZ and a p53-encoding plasmid to glioma cells. Our research had elucidated the circadian oscillations of the clock genes in the U87 glioma cells by employing two different computational models and observed that T16 and T8 time points revealed the highest circadian activity for *Bmal1* and *Per2* genes, respectively. Similar analysis was conducted for the transferrin receptor, which revealed that T7 and T8 were the key time points for its expression. A confocal microscopy study indicated the highest intracellular uptake of complexes and p53 mRNA expression at T8, the time point with the highest Per2 and transferrin receptor expression. Following mRNA analysis, the evaluation of p53 levels confirmed transcriptional changes at the protein level, and that T16 appears to be a favourable time point for enhancing therapeutic efficacy in U87 glioblastoma cells. These findings suggested that synchronizing the complexes’ administration with the biological clock of GBM cells may significantly improve glioblastoma therapeutics.

## 1. Introduction

Chronotherapy is an emerging strategy to improve cancer treatments by timing drug administration to a patient’s circadian rhythms, resulting in fewer side effects associated with higher efficiency and survival rates [1,2,3,4]. Circadian rhythms, commanded by the master clock in the suprachiasmatic nucleus (SCN) and peripheral clocks in cells, repeat daily, according to the presence or absence of certain environmental cues and influence diverse biological processes, including cell-cycle regulations and metabolism [1,3,4,5]. These rhythms are maintained by transcription–translation feedback loops (TTFL) involving core clock genes (as the basic helix-loop-helix ARNT-like 1 (Bmal1) protein and period circadian regulator 2 (Per2)), which modulate on their own and downstream gene expression [1,4,5].

Several studies associated the circadian rhythm disruption with the prevalence of cancer, namely glioblastoma, a grade 4 astrocytoma and the most prevalent and invasive form of primary brain tumour [5,6]. Benign brain tissue has the ability for invasion and rapid proliferation as resistance to apoptosis results in its high aggressiveness and poor prognosis with a median overall survival of 15 months despite current therapies such as surgery, radiation therapy, and/or lastly chemotherapy [5,6,7,8]. TMZ, the standard chemotherapeutic agent for glioblastoma, has shown limited efficacy, particularly in the patients lacking O6-methylguanine-DNA methyltransferase (MGMT) promoter methylation or isocitrate dehydrogenase (IDH) mutations [7,9,10]. The patients’ mutant for IDH whose tumours exhibit MGMT promoter methylation are associated with a better response to therapy [9,11]. However, the patient’s age, total tumour surgical resection impossibility, delay in radio chemotherapy, resistance to drug therapy associated with tumour heterogeneity, invasiveness, and abundant vascularity result in rapid recurrence and poor outcomes in the long term [7,10,12]. Additionally, the restrictive nature of the blood–brain barrier (BBB) further compromises outcomes [8]. Interestingly, recent studies suggest that glioblastoma cells retain functional circadian networks, and therapeutic responses, such as to TMZ, may depend on the timing of drug administration in relation to clock gene expression [5,13,14,15]. Then, a relationship has been established between circadian rhythm disruption and glioblastoma, providing an opportunity for chronotherapy [16,17].

In parallel, gene therapy and nanotechnology have been revolutionizing glioblastoma treatment by enabling precise drug delivery and gene modulation across the BBB, improving therapeutic efficacy while reducing side effects and the problem of drug resistance [18,19,20,21,22,23,24]. The restoration of tumour suppressor genes, such as TP53, has been explored to overcome resistance mechanisms in glioblastoma [7,25]. Nanocarriers, including cell-penetrating peptides (CPPs), offer a promising platform to deliver genes to tumour cells due to their biocompatibility, stability, ability to enhance drug stability and intracellular delivery, and potential for surface functionalization to reduce off-target toxicity and achieve tumour targeting [21,26]. CPPs are composed of less than 30 amino acids, have an overall positive charge, a remarkable ability to carry out different molecules, self-assemble and cross cellular membranes in a non-invasive manner [27,28]. Several studies have highlighted the potential of CPPs-based carriers for glioma treatment [29,30,31].

Building on this foundation, our research group previously developed WRAP5 CPP-based nanocomplexes functionalized with a transferrin (Tf) ligand and co-loaded with TMZ and a TP53-encoding plasmid DNA [29,32]. These complexes proved to be biologically compatible to several cell lines and for zebrafish (*Danio rerio*) embryos, and effective in facilitating targeted delivery and activating the intrinsic apoptosis pathway in a wild-type p53 (U87) and two mutant p53 (SNB19 and U373) cell lines [29,32]. However, the influence of circadian timing on their performance remained unexplored.

This study investigated how the glioblastoma cells’ circadian rhythms affect the delivery of complexes TMZ/*TP53* and p53 gene/protein expression. By analyzing the oscillations of clock-related genes (*BMAL1* and *PER2*) and transferrin receptor levels in U87 glioma cells, we identified optimal timing for the complexes’ cellular uptake and payload delivery using two complementary computational tools (CircWave and CosinorPy). Then the complexes’ ability to increase intracellular p53 levels at these time points were evaluated. Our findings strongly suggest that the consideration of the cell’s intrinsic circadian rhythm and the selection of a specific time point in the TMZ/TP53 systems’ delivery approach may enhance the systems’ cellular targeting, uptake and, ultimately, therapeutic precision in glioblastoma treatment.

## 2. Results and Discussion

### 2.1. Bmal1, Per2, and Transferrin Gene Expression Analyzed by CircWave

**Figure 1:**
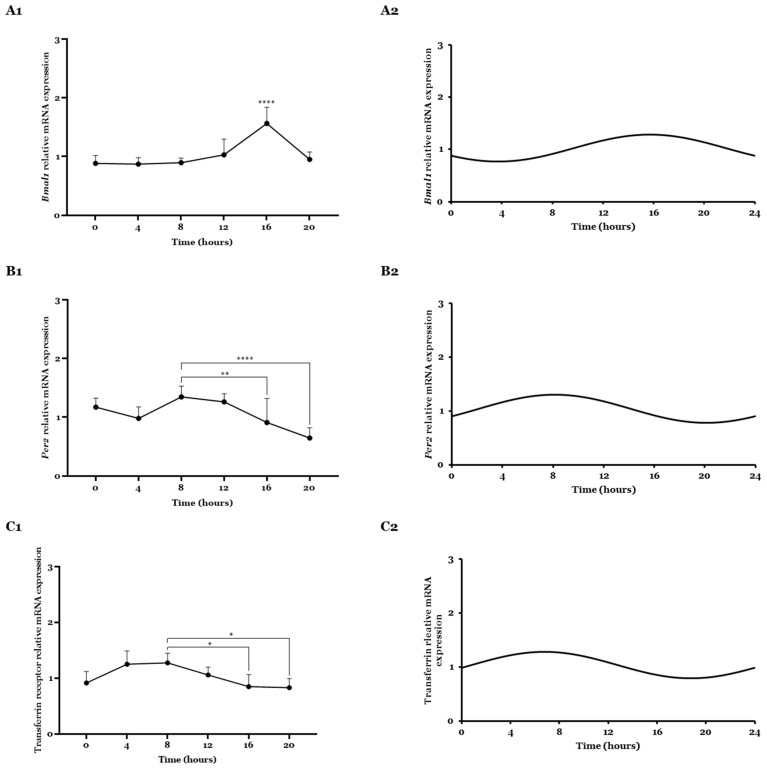
Relative mRNA expression profiles of core clock and transferrin receptor gene. (**A1**,**B1**,**C1**) Expression profile of *Bmal1*, *Per2,* and transferrin receptor gene in U87 cell line over 24 h. To calculate the ΔCt mean value, the average of Cts of the housekeeping gene for all samples was subtracted from the average of Cts of all gene samples. Then, ΔΔCt values were calculated by subtracting the ΔCt mean value from the ΔCt of each sample. Mean ± SD are shown (n = 6). (**A2**,**B2**,**C2**) Data was analyzed by one-way ANOVA followed by the Bonferroni test. Circadian oscillations are statistically significant during a 24 h period as analyzed with CircWave (*p* < 0.05). Statistical analysis is shown in Table 1. *, *p* ≤ 0.05; **, *p* ≤ 0.01; ****, *p* ≤ 0.0001.

Analyzing mRNA expression patterns of core clock genes via RT-qPCR is still one of the most common strategies to study circadian cell rhythms [33]. Therefore, *Bmal1* and *Per2* clock-related genes’ circadian expression was assessed by RT-qPCR every 4 h for 24 h. Figure 1 displays the obtained results for the *Bmal1*, *Per2* genes, as well as for the transferrin receptor gene.

From the plot analysis (Figure 1(A1)), a variation in *Bmal1*’s relative mRNA expression over time was observed, with certain time points representing higher and lower values of expression. Comparing the different time points with each other, T16 appeared as the time point with statistically significant higher *Bmal1* expression over the 24 h cycle compared with all the other time points (***, *p* ≤ 0.0001). Circadian oscillation pattern was statistically significant after CircWave analysis as defined by a *p* < 0.05 (Figure 1(A2), Table 1). A centre of gravity (COG), which defines when circadian activity is tendentially centred, corresponds to the T16 time point, which agreed well with the time when more mRNA expression was observed (Figure 1(A1)).

The *Per2* relative mRNA expression and rhythmicity were also investigated (Figure 1(B1,B2)). Figure 1(B1) shows the variation in *Per2* mRNA expression over time. After comparing statistically, the different time points, T8 appeared as the time point with higher mRNA expression, regarding the statistical significance difference with T16 (**, *p* ≤ 0.01) and T20 (****, *p* ≤ 0.0001) time points. CircWave analysis indicated rhythmicity of data as indicated by *p* < 0.05 (Figure 1(B2), Table 1). A COG agreed well with the mRNA expression profile observed for *Per2* corresponding to the T8 time point.

Transferrin, a blood–plasma glycoprotein responsible for maintaining the supply of iron ions during cell growth with a high demand for iron, through a TfR-mediated transcytosis, has been widely used to functionalize nanoparticles for brain cell delivery [29,32,34,35,36]. Moreover, this receptor has been described as overexpressed in glioma cells up to 100-fold higher compared to healthy cells and with a higher malignancy grade [34,37,38,39]. As the described TMZ/WRAP5/pDNA complexes were functionalized with a transferrin ligand (Tf-WRAP5), it seemed of extreme importance to study the receptor expression over the same time, and also, evaluate its rhythmicity. Figure 1(C1,C2) shows the obtained results. Figure 1(C1) presents the variation in TfR relative mRNA expression over 24 h. Similar to *Per2*, higher expression was noticed for T8 time point, with statistical significance with T16 (**, *p* ≤ 0.01) and T20 (****, *p* ≤ 0.0001) time points. Additionally, TfR expression is circadian regulated as proved by CircWave analysis (*p* < 0.05) (Figure 1(C2), Table 1). A COG around T7 was obtained. These two metrics do not always coincide, since the COG obtained value is influenced by the overall distribution of mRNA expression levels over the day, and the peak of mRNA expression is determined by that exact time of quantification. In other words, the average phase of gene expression does not necessarily coincide with the time of maximum expression [40,41]. Notwithstanding being performed in other cell lines, TfR has already been shown to have a significant 24 h rhythm.

**Figure 2:**
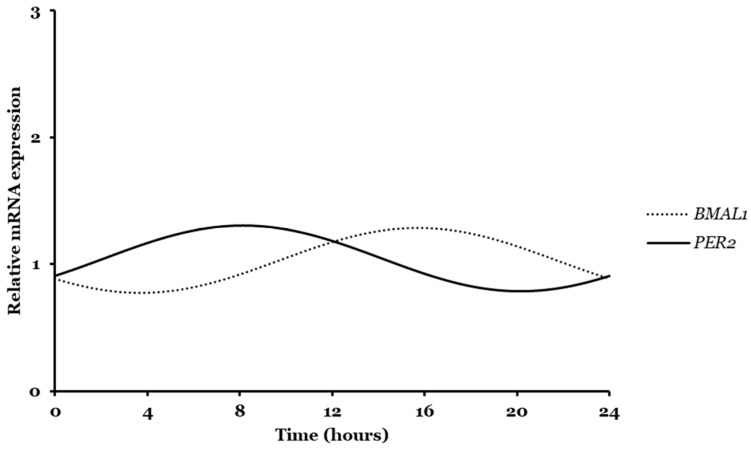
Circadian oscillations of *Bmal1* and *Per2* genes in U87 cell line over 24 h. Data graphics were obtained after CircWave analysis.

It was also particularly relevant for authors to compare circadian expression patterns of both *Bmal1* and *Per2* genes, as this could provide proof of the feedback loop involved in the circadian rhythm of glioma cells. Figure 2 represents the overlay of sinusoidal curves obtained after CircWave analysis for *Bmal1* and *Per2*.

As hypothesized, an antiphase expression profile with a 12 h phase difference was observed (Figure 2). It is worth highlighting that the analysis of Table 1 already showed this result of 12 h activity phase difference between the COG value of *Bmal1* (T16) and *Per2* (T8). When *Per2* transcription is activated by CLOCK/Bmal1 dimer, it translocates to the nucleus and suppresses its own transcription by inhibiting Bmal1 expression [4]. Similar results with antiphase expression patterns of Bmal1 and Per2 were found for MCF19 breast cancer cells, paraventricular nucleus (PVN) cells, and human GBM cells [13,33,42].

**Figure 3:**
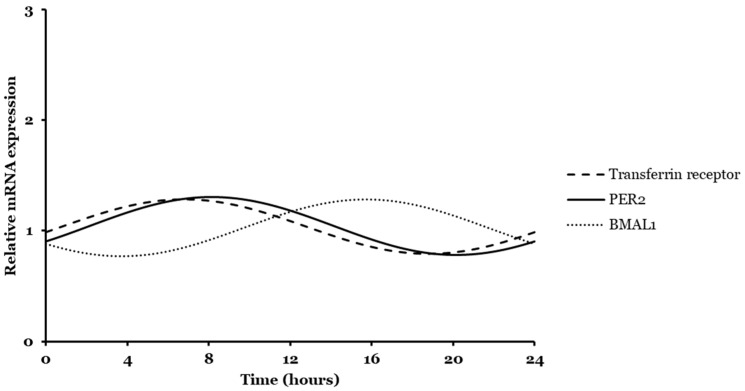
Circadian oscillations of *Bmal1*, *Per2*, and transferrin receptor genes expression over 24 h. Data graphics were obtained after CircWave analysis.

Moreover, it was valuable to explore the connections between *Bmal1* and *Per2* clock-related genes and the TfR gene. Despite the few published work, there is some evidence of Bmal1 interaction with circadian locomotor output cycles kaput (CLOCK) to regulate the expression of some iron regulatory proteins, such as transferrin receptor 1 (TfR1), in an indirectly rhythmical way. Knockdown of *Bmal1* in human trophoblast (HTR8) and epithelial JEG-3 cells led to changes in the iron-dependent oxidative stress mechanism, altered ferroptosis-related proteins expression and increased TfR1 expression [43]. However, another study using a U252 glioma cell line altered to endogenously overexpress TfR1 (U251-TfR1), linked the overexpress of TfR1 to elevated cellular iron levels and reduced expression of Bmal1 and increased expression of Per2, suggesting that core clock genes and TTFL feedback loop may be regulated by cellular iron levels in the brain [44]. Authors speculated that the effect of iron levels on the TTFL would initiate on Per1 protein levels modulation [44]. Another study, in mice colon-26 cell line xenografts, transferrin receptor regulation was shown to have 24 h circadian rhythmicity influenced by the rhythmical expression of the iron regulatory protein 2 (IRP2), which transcription is in turn promoted by Bmal1 binding to *IRP2* gene at E-box [45,46]. Contrary to the previous study, these findings suggest that Bmal1 may influence the post-transcriptional regulation of TfR1, potentially leading to increased receptor protein levels and elevated intracellular iron content. An interesting experiment carried out by Pasricha et al. with liver and bone marrow of mice revealed circadian oscillations of TfR1 gene following oscillations of serum iron across the day [47,48]. Indeed, from our CircWave data, the TfR circadian oscillation pattern is more coincident with the *Per2* pattern than with *Bmal1*, as demonstrated in Figure 3. Actually, we observed a similar antiphase rhythmic expression pattern as previously for *Bmal1* and *Per2* comparison (Figure 2). For relative mRNA expression, a higher peak activity happens almost simultaneously (around T7). We hypothesized that TfR displays a circadian rhythm, even if it is in an indirect way and it is positively influenced by Per2 activity and Bmal1 repression. In summary, Bmal1 and Per2 may indirectly regulate iron metabolism, thereby affecting TfR expression. Increased receptor-mediated endocytosis is likely to occur during periods of reduced Bmal1 and elevated Per2 expression, suggesting a circadian window of enhanced cellular uptake capacity. For further conclusions, future studies should be conducted using U87 glioma cells regarding the iron content in cells and its rhythmicity.

### 2.2. Bmal1, Per2, and Transferrin Gene Expressions Analyzed by CosinorPy

Behind the main advantages displayed by CircWave software as the determination of peak phase or the centre of gravity (COG), some disadvantages include the impossibility of analyzing circadian data beyond a 24 h period and the fact that this model is limited regarding the recognition of tooth-shaped curves and spiky forms [49]. Then, its sensitivity to recognize rhythms might be affected [49]. To overcome these issues, a necessity to explore a novel computational approach emerged. CosinorPy, a Python software package developed by Miha Moškon’s group, allowed for the detection and analysis of circadian rhythmicity of data related to *Bmal1* and *Per2* relative mRNA expression for longer periods. The cosinor method was developed to overcome some non-parametric methods and is based on a trigonometric regression model (Equation (1)) [50,51]. The advantages of using this method include the possibility to apply a multi-component cosinor regression to data and to be executed in Python, one of the most suitable programming languages for beginners [50]. Several other studies have been implementing this computational approach for cells circadian rhythms analysis [52,53,54]. Accordingly, a cosinor module was applied to the data to generate synthetic information for analysis. This module has functionalities as model fitting, comparison between models to decide which one fits better, and analysis of different rhythmic parameters as fitting statistical significance (*p*-value), MESOR (M), amplitude (A), and acrophase (Ø) [50]. MESOR refers to a midline of estimated rhythm over time, amplitude is a measure of half of the predicted change extension, and acrophase is the time of peak activity, similar to COG in CircWave analysis [55].

**Figure 4:**
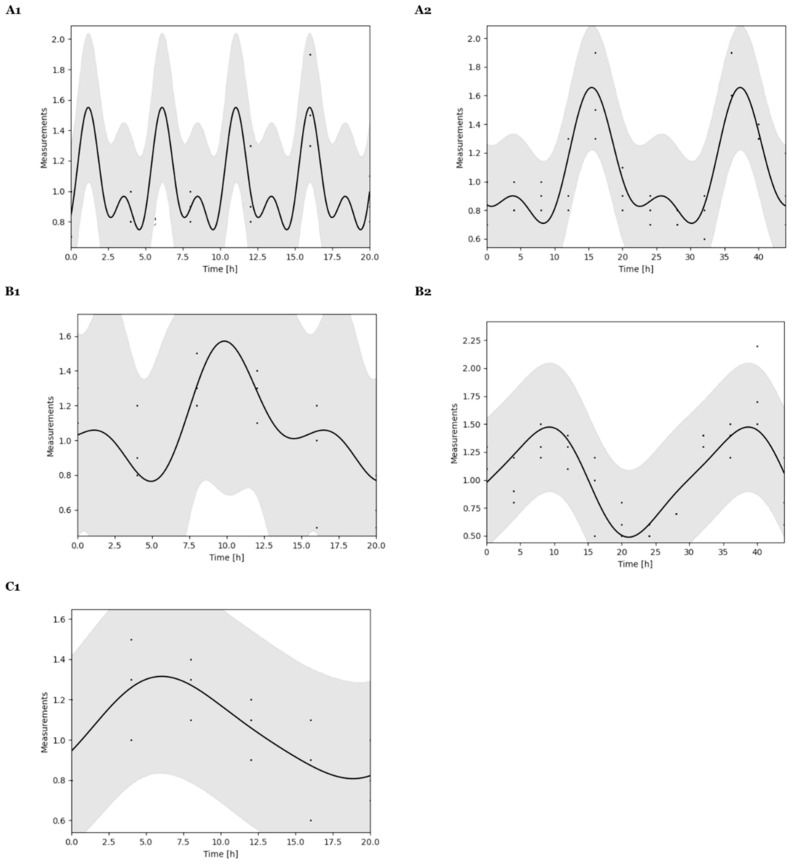
Differential rhythmicity analyses of core clock and transferrin receptor genes. Circadian curve profile of *Bmal1* (**A**), *Per2* (**B**), and transferrin (**C**) gene performed with the single component cosinor analyses, for 24 h (**1**) and 48 h (**2**).

Initially, a comparison between single and multi-component cosinor models allowed for the choice of the best model fitting to data (Supplementary Materials, Results Section, Table S3). The Table summarizes the values of Residual Sum of Squares (RSS) and Mean Squared Errors (MSE) calculated for both models and for each gene studied. The same values of RSS and MSE suggested that both models can satisfactorily predict our 24 h and 48 h period data analysis. Afterwards, a single component cosinor model was applied to analyze qPCR data collected at both 24 h and 48 h. Figures regarding time series rhythmicity were automatically generated (Figure 4) as the parameters were estimated (Table 2).

For 24 h data, results presented in Figure 4(A1,B1,C1) and Table 2 showed a general good fit of the chosen model to the dataset. A *p*-value equal to or under 0.05 confirmed the adequacy of the cosinor model and indicated statistically significant rhythmicity [50]. Miha Moškon’s group analyzed different rhythmic data and proved that CosinorPy methodology can assess statistics of rhythmicity, even in cases where the observed data exhibit various asymmetric peaks within a single rhythm period [50,51]. Thereby, this model was able to accurately reproduce *Bmal1* and *Per2* relative mRNA expression oscillatory signals, but not for the transferrin gene, within the periodicity calculated (Table 2). It seemed that these models were only adequate for *Bmal1* and *Per2* gene analysis. Even though the rhythmic pattern does not show a statistically significant periodicity for the transferrin receptor gene, other statistical models can be tested for a curve fit in the future. This result can just be derivate of limitations of the model that is not able to detect its rhythmicity or insufficient data. For this case, beyond CircWave, Circa Diem MATLAB toolbox, CircadiPy, and GLMMcosinor are examples of other tools that could be used for cosine model’s application and transferrin receptor circadian rhythm’s parametric analysis [56,57,58].

CosinorPy was additionally used to identify rhythmicity and related parameters for 48 h dataset of *Bmal1* and *Per2* genes. Figure 4(A2,B2) and Table 2 show and summarize the data generated. Results suggested a perfect and accurate fit of the model to the data (Table 2). For *Bmal1* and *Per2*, *p*-values below 0.05 indicated that the model was able to adequately describe rhythms and accurately reproduce the oscillatory signal.

Both CircWave and CosinorPy package can be used to assess the statistics of the rhythmicity parameters using qPCR data. Both tools are able to provide information regarding the rhythmicity of genes under study. However, trigonometric regression models seemed more accurate in calculating parameter statistics. As evidenced by the *p*-value for 48 h data, the acquisition of more points provided much richer statistics.

### 2.3. Transferrin Receptor Expression

**Figure 5:**
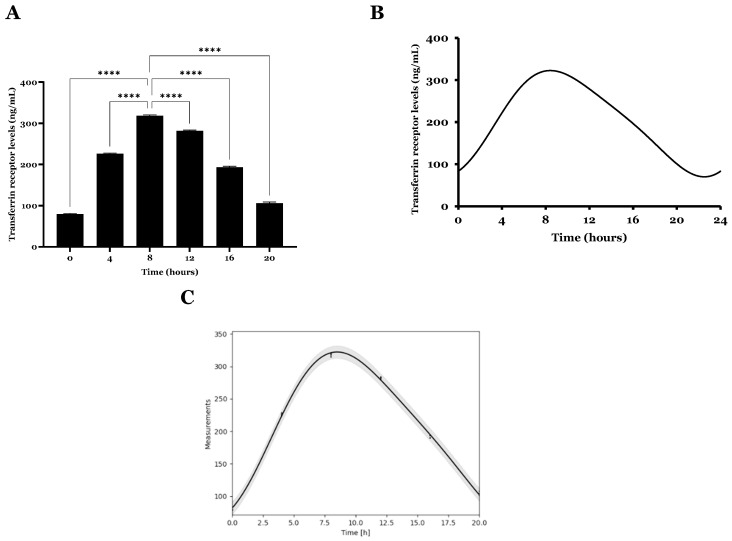
Transferrin receptor expression and circadian pattern in U87 cell line over 24 h. (**A**) Expression profile of transferrin receptor levels. Mean ± SD are shown (n = 3). Data was analyzed by one-way ANOVA, followed by the Bonferroni test. Statistical significance (****, *p* ≤ 0.001) was found between all time points. (**B**) Differential rhythmicity analysis of TfR expression over 24 h performed with CircWave. (**C**) Differential rhythmicity analyses of TfR expression over 24 h were performed with the single component cosinor model.

In addition to transcript quantification, TfR expression was quantified in U87 cells through an ELISA immunoassay over 24 h. The TfR levels for each time point are presented in Figure 5A. The CircWave and cosinor models were also utilized to assess the receptor rhythmicity and associated parameters in the 24 h dataset. Results are presented in Figure 5B,C.

The quantification revealed that the TfR content varied over 24 h. In agreement with mRNA expression, a high content was detected for T8 when compared to the other time points (****, *p* ≤ 0.0001). From our data, it also became clear that the TfR expression presents a 24 h circadian oscillation. From our knowledge, results from circadian rhythm analysis suggested that the CircWave model is better adequate for data, with a *p*-value under 0.05 (Table 3). This result agreed well with the previously presented findings of relative mRNA expression and CircWave analysis. The variation in mRNA levels seemed to influence subsequent TfR content variation in glioma cells [46]. CircWave suggested a centre of gravity around T10 for the TfR expression, a slightly high value compared to the mRNA transcripts (T7). This can be due to the biological timing that occurs between gene transcription in the nucleus and protein translation in the cytoplasm of glioma cells.

### 2.4. Cellular Internalization and Complexes’ Co-Localization

**Figure 6:**
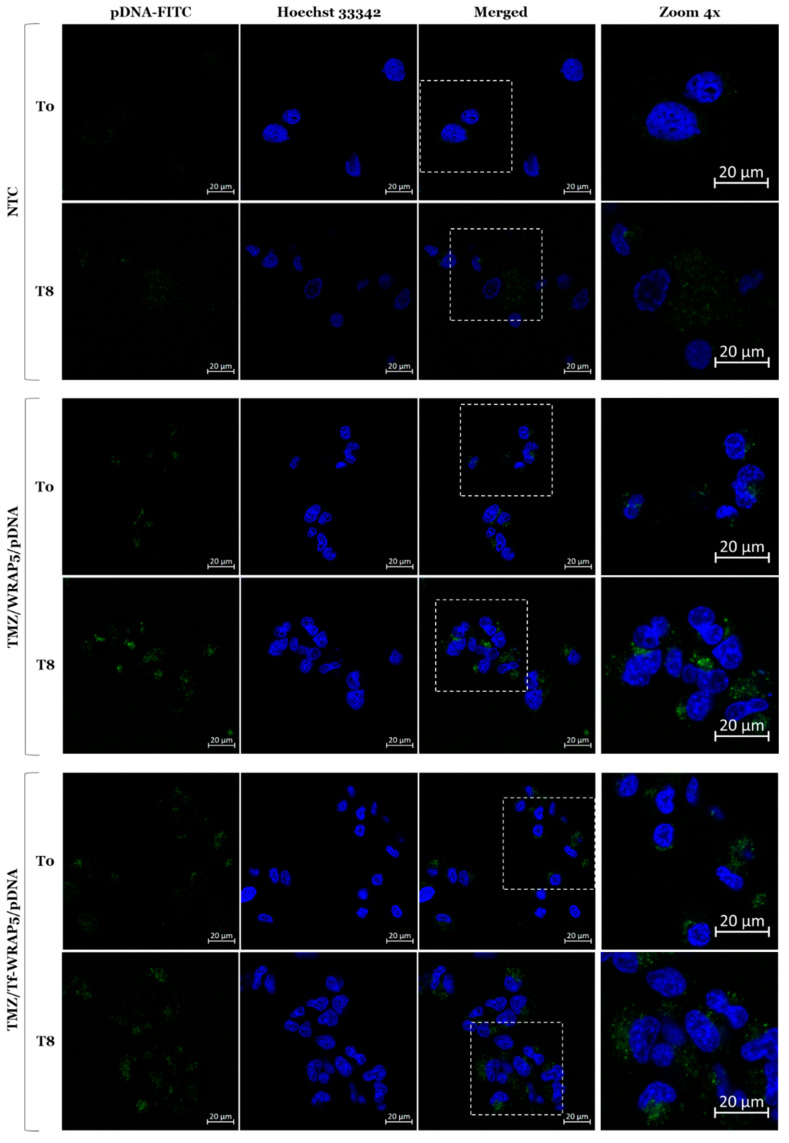
Fluorescence confocal microscopy study. Representative live cell images of U87 cell line after 1 h of transfection mediated by the different TMZ/WRAP5/pDNA-FITC complexes developed at N/P ratio 1 (using 0.1 µg pDNA). Cells were transfected with complexes at T0 and T8 time points. Hoechst 33342 dye stained the nuclei (blue) while FITC dye stained pDNA (green). NTC—non-transfected cells. Scale bar = 20 µm.

After the deep analysis of transferrin receptor levels and respective rhythmicity, specific time points were chosen for a fluorescence confocal microscopy study in U87 cells. The idea was to monitor the differences in developed TMZ/WRAP5/pDNA-FITC complexes’ internalization effectiveness into glioma cells between those time points. Experiments were performed in a live cell approach at T0 and T8 time points, time points associated with the lowest and highest transferrin receptor expression. As described in the Methods Section 3.2, Hoechst 33342 probe solution was used to stain nuclei (blue), and pDNA was stained by FITC (green). Representative U87 cells’ fluorescent images are displayed in Figure 6.

Non-transfected cells (NTC) were considered as the control, as demonstrated by the lack of detectable fluorescence. The two subsequent sets of images, corresponding to U87 cells transfected with TMZ/WRAP5/pDNA-FITC complexes at both time points proved the intracellular uptake of the complexes, with pDNA-FITC localized in the cytoplasm and perinuclear region of glioma cells. Additionally, fluorescence was detected within the nucleus, as observed in the merged image. At T0 time point, the pDNA-FITC fluorescence signal seemed to have a substantial difference between complexes not bearing (TMZ/WRAP5/pDNA-FITC) and bearing the Tf ligand (TMZ/Tf-WRAP5/pDNA-FITC). Probably, due to lower levels of TfR at the glioma cell surface at this time point, functionalization of complexes appeared even more important and increased internalization success. This result supports the importance of the Tf ligand presence as it enhances cellular entry by binding to TfR, enabling efficient intracellular delivery via receptor-mediated endocytosis. These observations agreed well with previously documented data on U87 cells [29,32]. At the T8 time point, data did not appear to show a difference in internalization ability between the two types of complexes. We speculated that because of higher receptor expression, the presence of ligand at the complexes surface and its effect may fade away, and both complexes are perfectly taken up by cells. Nonetheless, a remarkable difference was found for pDNA-FITC staining between T0 and T8 time points for both complexes. Internalization occurred to a greater extent at the T8 time point, as expected by the higher levels of TfR at the cell surface. This result further supports the proposed link between Bmal1/Per2 expression patterns and receptor-mediated endocytosis. Specifically, the temporal correlation between Per2 upregulation, Bmal1 suppression, and increased transferrin receptor activity suggests that circadian clock components may modulate the endocytic efficiency of WRAP5/pDNA complexes.

**Figure 7:**
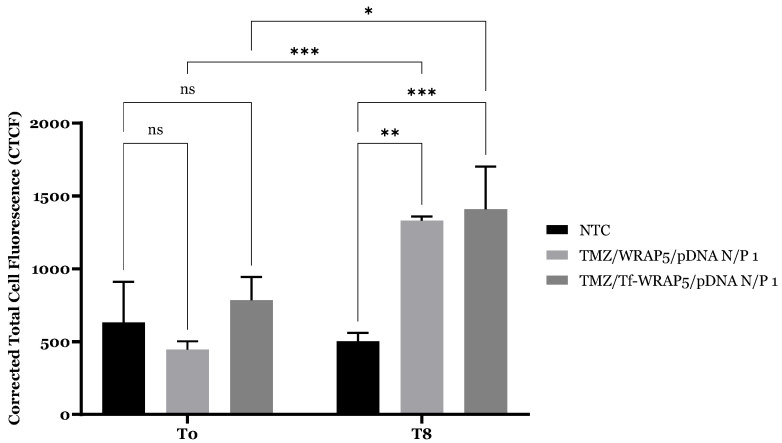
Corrected Total Cell Fluorescence (CTCF) profile of U87 cell line. U87 were transfected with TMZ-bearing complexes developed at N/P ratio 1 (using 0.1 µg pDNA) at T0 and T8 time points. Mean ± SD are shown (n = 3). Data was analyzed by two-way ANOVA followed by the Bonferroni test. ns, *p* > 0.05, *, *p* ≤ 0.05; **, *p* ≤ 0.01; ***, *p* ≤ 0.001.

Additionally, Corrected Total Cell Fluorescence (CTCF) was obtained after fluorescence quantification of acquired microscopy images using ImageJ (https://imagej.net/ij/). Figure 7 summarizes the results obtained for complexes at T0 and T8 time points in U87 cells. At T0, there was no statistically significant difference between the TMZ/Tf-WRAP5/pDNA-FITC and TMZ/WRAP5/pDNA-FITC complexes CTCF compared to the control, indicating minimal cellular uptake of complexes at this time point. At T8, quantification data revealed a more efficient cellular uptake for TMZ/Tf-WRAP5/pDNA-FITC complexes (***, *p* ≤ 0.001) than for TMZ/WRAP5/pDNA-FITC complexes (**, *p* ≤ 0.01) when compared to non-transfected cells. The functionalization of the complexes with the Tf ligand facilitated penetration of CPP-based complexes into glioma cells, as predicted. In the end, a statistically significant difference in internalization of complexes between the two time points (***, *p* ≤ 0.001 for not bearing Tf complexes and *, *p* ≤ 0.05 for Tf bearing complexes) was observed, supporting the previously discussed results of the fluorescence confocal microscopy study.

Altogether, this set of observations underlined the importance of taking into consideration the circadian profile of cell surface receptors as it may improve nanocomplex uptake, delivery performance, and, in the end, therapeutic effect.

### 2.5. TP53 and p53 Expression

**Figure 8:**
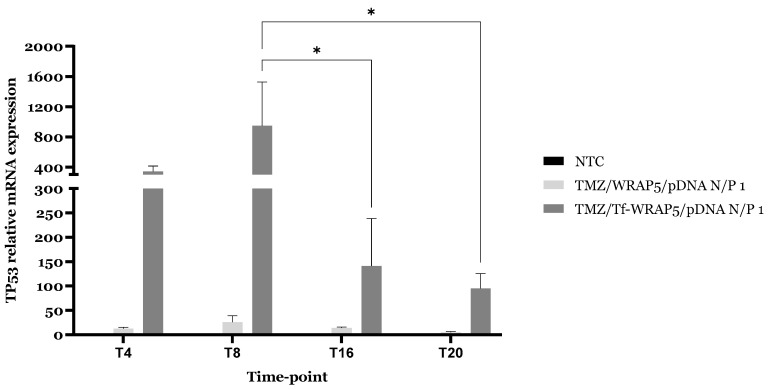
Relative mRNA expression profiles of the *TP53* in U87 cell line. Transfection was mediated by the different WRAP5/pDNA-FITC complexes developed at an N/P ratio of 1 (using 1 µg pDNA) at T4, T8, T16, and T20 time points. To calculate ΔCt mean value, the average of Cts of the housekeeping gene for all samples was subtracted from the average of Cts of all gene samples. Then, ΔΔCt values were calculated by subtracting ΔCt mean value from the ΔCt of each sample. Mean ± Standard Error of the Mean (SEM) are shown (n = 6). Data was analyzed by one-way and two-way ANOVA, followed by the Bonferroni test. *, *p* ≤ 0.05.

Quantification of the *TP53* expression in U87 cells at the designated time points allows us to evaluate the effectiveness of TMZ/WRAP5/pDNA complexes for cellular internalization and gene delivery/expression, and consequently, the potential to induce therapeutic effect. p53 cell expression levels were previously discussed after nanocomplex transfection, but without considering glioma circadian rhythm [32]. Developed complexes were capable of inducing a significant increase in protein cytoplasm content in comparison with non-treated cells (****, *p* ≤ 0.0001) [32]. A successful delivery of cargo, a plasmid coding for p53, culminated in a higher protein production. In the present work, the authors investigated the impact of the circadian rhythm on gene expression, and on the prospective therapeutic effect, of the developed complexes bearing, or not, the transferrin ligand. The time points chosen were the same as the intracellular co-localization experiments, so a deep discussion concerning the influence of receptor circadian oscillations in the *TP53* could take place. p53 relative mRNA expression was monitored by RT-qPCR, and the results are presented in Figure 8.

Figure 8 demonstrates that p53 mRNA content varied within the complexes applied for treatment and within the time points. Both TMZ/WRAP5/pDNA complexes were able to induce an increase in mRNA content after transfection compared to non-transfected cells, but complexes bearing transferrin ligand showed improved results with higher expression extent compared to TMZ/WRAP5/pDNA complexes. This proved the importance of complex functionalization for a more efficient cell uptake and internalization, and later p53 mRNA transcripts production. Within the time points, data indicated that p53 mRNA expression peaks at T8 following carrier transfection, with statistically significant differences compared to T16 and T20 (*, *p* ≤ 0.05). Another important observed fact was the coincidence of time point with higher TfR expression (T8) with the time point with higher p53 mRNA expression (T8). Higher expression of the cell surface receptor enhanced the internalization of functionalized complexes and transfection success, which in turn improved pDNA delivery to the nucleus. This ultimately leads to more efficient gene expression.

**Figure 9:**
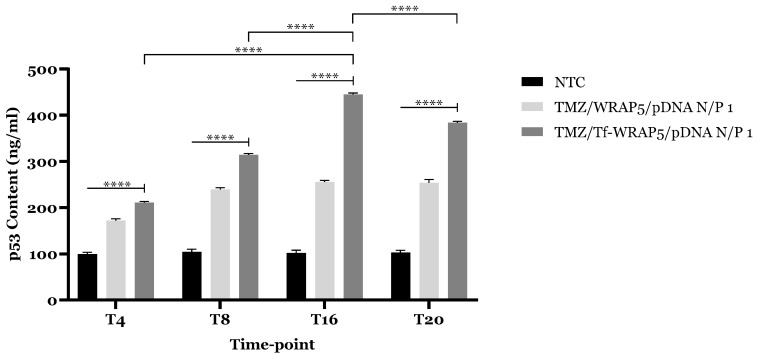
p53 levels in U87 cells after 24 h of transfection mediated by the different WRAP5/pDNA complexes developed at N/P ratio of 1 (using 1 µg pDNA) at T4, T8, T16, and T20 time points. Mean ± SD are shown (n = 3). Data was analyzed by a two-way ANOVA, followed by the Bonferroni test. ****, *p* ≤ 0.0001.

In addition, previous studies have found a linkage between Per2 and Bmal1 clock-related proteins and p53 pro-apoptotic protein expression, stability, and activity [59,60,61,62,63]. According to several studies, Per proteins seemed to bind to *Per2* promoter and prevent p53 degradation and ubiquitylation by MDM2 (E3 ubiquitin-protein ligase), a natural negative regulator of p53 activity, to maintain basal levels of p53 under stressful conditions [59,60,61,64]. It is also known that overexpression of Per2 was linked to increased levels of p53 and Bmal1 downregulation induced p53-dependent p21 expression [59,61]. Contrarily, Bmal1/Clock complex promotes the de-acetylation of p53 for DNA repair and apoptosis inhibition [59,61]. In an in vivo xenograft model, downregulation of Bmal1 and Per2 also seemed to influence the circadian expression profile of the *TP53* [65]. So, clock-related proteins, particularly those involved in the regulation of circadian rhythms, can significantly influence the expression and activity of the *TP53* and its associated protein throughout the cell cycle. Then, quantifying p53 after analyzing its mRNA expression by RT-qPCR is especially important due to the p53 time-dependent regulation and post-transcriptional mechanisms that occur within the circadian rhythm influence. Therefore, for each time point, protein expression was quantified in U87 cells 24 h post-transfection mediated by the complexes, through a p53 ELISA immunoassay. Figure 9 represents the p53 levels obtained at each time point and for each delivery system investigated. As observed, all WRAP5/pDNA complexes were able to induce a statistically significant increase in intracellular p53 levels at all four time points compared to non-transfected controls (****, *p* ≤ 0.0001). Notably, the extent of p53 expression varied depending on the type of complex used in cellular transfection. TMZ/Tf-WRAP5/pDNA complexes were associated with higher p53 content regarding the TMZ/TWRAP5/pDNA complexes. This finding is consistent with previous data, suggesting that the presence of the transferrin ligand enhances complexes’ performance. Furthermore, p53 content varied within the time points considered. Data indicated higher protein production at T16 in comparison with the other time points (****, *p* ≤ 0.0001). This finding indicates that T16 may represent the most favourable time point for achieving maximal therapeutic efficacy in U87 glioblastoma cells.

It is well-established that a phase delay often exists between the peak of mRNA expression and the corresponding protein peak levels, primarily due to the time required for mRNA translation and protein accumulation [66,67,68]. This phenomenon is evident in the current study, where data from Figure 9 demonstrated a delayed increase in p53 content relative to the peak in p53 mRNA expression observed in Figure 8. Specifically, the highest mRNA levels were recorded at the T8 time point, while maximal p53 accumulation occurred at T16. Such temporal discrepancy is consistent with phase shifts between transcription, translation, and protein stabilization that can be under circadian rhythm effects. This also suggests that T16 may be an optimal time for maximizing the therapeutic effect in U87 glioblastoma cells. It would be valuable to investigate p53 levels at time points between T8 and T16, T12 for instance, as T12 may be the time point corresponding to the onset of protein translation, as it is hypothesized that this phenomenon may occur early. Additionally, evaluating BMAL1 and PER2 protein expression patterns at these specific time points could provide insights into potential temporal regulatory interactions with p53.

This data further reinforces the importance of incorporating circadian rhythms into gene therapy protocols in order to enhance the nanoparticle-based drug/gene delivery systems therapeutic performance in cancer treatment. This chronobiological approach offers a promising strategy for improving the precision and effectiveness of gene-based therapies, particularly in highly resistant malignancies such as glioblastoma.

Nevertheless, this study has some limitations that should be acknowledged. Our findings are based exclusively on the U87 glioma cell line, which may not fully represent the molecular heterogeneity of glioblastoma. Additionally, the study focused on transcript-level analyses without corresponding protein-level results, which is important given the potential for circadian post-transcriptional regulation. To address these limitations, future studies will include the assessment of Bmal1 and Per2 protein intracellular levels at the studied time points, p53 expression and other apoptosis-related proteins, including caspases 3/9 and Bcl-2 Associated X-protein (Bax), and the study of other circadian rhythm regulators in GBM, such as Cry1/2, at both transcript and protein levels. We also plan to assess chronotherapy validation in other GBM cell lines (such as SNB19 and U373, already studied by the lab group) and 3D multi-cell culture models. From a clinical translational perspective, future studies should consider in vivo models that more accurately replicate the circadian physiology of glioblastoma and drug distribution and toxicity. *Bmal1* and *Per2* circadian gene knockout (e.g., CRISPR/Cas9) and/or GBM patient-derived xenograft models (zebrafish and rodent models) could help to determine the optimal time points for complexes’ administration after studying its chronopharmacokinetic (absorption, distribution, metabolism, and excretion). These future directions aim to enhance the biological relevance of our findings and could ultimately contribute to the development of more personalized and effective treatment strategies for glioblastoma, a highly aggressive cancer with limited therapeutic options.

## 3. Materials and Methods

### 3.1. Materials

Synthesis of WRAP5 and Tf-WRAP5 peptides was performed in a synthesizer LibertyBlue™ Microwave Peptide (CEM Corporation, Matthews, NC, USA) coupled to a Discover™ module (CEM Corporation, NC, USA). The microwave energy at 2450 MHz to the Fmoc/tert-butyl (tBu) strategy was applied during the process. Peptides were lastly supplied lyophilized (~95% purity (HPLC/MS)) and preserved in the fridge for future use.

The plasmid pcDNA3-FLAG-p53, 6.59 kbp (Addgene plasmid # 10838, Cambridge, MA, USA) was a gift from Thomas Roberts. The plasmid was produced, extracted, and purified by a protocol developed and already described in the literature by our research group [69]. The p53-encoding plasmid was prepared from the same batch for all transfection experiments to ensure consistency. The TMZ drug was purchased from Cayman Chemical (Item No. 14163, ≥98% purity, Lisbon, Portugal) and Dulbecco’s Modified Eagle’s Medium (DMEM) high glucose with stable L-Glutamine from Biowest (L0103, Nuaillé, France). The dye fluorescein isothiocyanate (FITC), isomer 1, was purchased from Sigma Aldrich Chemicals (F7250, ≥90% purity, St. Louis, MO, USA) and Hoechst 33342, trihydrochloride trihydrate, from Invitrogen (H1399, LOT 1932847, ≥99% purity, Waltham, MA, USA). Transferrin Elisa kit (No-EH0669) was purchased from Universal Biologicals (Cambridge, UK) and the p53 ELISA kit from Roche (Roche Applied Science, Penzberg, Germany). All solutions were prepared using Milli-Q ultrapure grade water (Billerica, MA, USA).

Human brain-like glioblastoma U87 (wild type p53) cell line was obtained from the American Type Culture Collection (ATCC^®^, Manassas, VA, USA) as a gift from Professor Jorge Lima (Faculty of Medicine of the University of Porto and IPATIMUP).

### 3.2. Methods

#### 3.2.1. Cell Culture

For in vitro experiments, U87 cells were cultivated in DMEM using high glucose with stable L-glutamine medium supplemented with fetal bovine serum (FBS) 10%, and 1% (*v*/*v*) of a mixture of penicillin (100 µg/mL) and streptomycin (100 µg/mL), at pH 7.40–7.45. Cells were kept growing at 37 °C in a humidified atmosphere containing 5% CO_2_ and sub-cultivated every 3 days to maintain their exponential growth and a regular metabolism.

#### 3.2.2. Cell Synchronization

U87 cells were seeded according to the experiment specifications and maintained at 37 °C with 5% CO_2_. On the 3rd day of culture, the culture medium was replaced by a fresh culture medium containing 0.1 µM dexamethasone, and cells were left for synchronization for 2 h. After synchronization, cells were returned to dexamethasone-free medium. The same procedure was conducted for all experiments. This method was already described and employed in several other cell lines for circadian rhythm studies by our research group [70,71].

#### 3.2.3. Rhythmic Oscillations of Bmal1, Per2, and Transferrin Receptor Gene Analyzed by Quantitative Reverse Transcription Polymerase Chain Reaction (RT-qPCR)

To analyze gene expression, U87 cells were seeded at a density of 10^5^ cells/well onto a poly-L-lysine coverslip 12-well plate and grown in 1.5 mL of medium. After synchronization, cells were collected every 4 h through a cycle of 24 h and 48 h for posterior RNA extraction (please consult Supplementary Materials, Methods Section, topic “*RNA Extraction*”). The first time point (T0) was defined as the moment immediately following cell synchronization, followed by subsequent time points at T4, T8, T12, T16, T20, and continuing at intervals of 4 h until the T44 time point. cDNA synthesis was carried out from 1 µg of pure extracted RNA using the NZY First-Strand cDNA Synthesis Kit (NZYtech, Lisbon, Portugal), following the manufacturer’s protocol. Conventional PCR amplification protocol of *Bmal1* and *Per2* cDNA, as well as primer sequences, are fully described in the Supplementary Materials, Methods Section, topic “*Bmal1 and Per2 gene as analyzed by conventional PCR*”. Human glyceraldehyde 3-phosphate dehydrogenase (GAPDH) was used as a housekeeping gene. The NZYSupreme qPCR Green Master Mix (2x) kit (NZYtech, Lisbon, Portugal) was used in RT-qPCR experiments for amplifying and quantifying core clock and transferrin genes, considering the manufacturer’s instructions. For amplification, samples were placed in a CFX-Connect^TM^ Real-Time PCR Detection System (Bio-Rad, Hercules, CA, USA) using a protocol consisting of an initial polymerase activation at 95 °C for 2 min, followed by a denaturation at 95 °C for 5 s, and a final annealing/extension step at 60 °C for 15 s for *Bmal1* and *Per2* genes and 20 s for transferrin studies. The amplification of the transcripts was confirmed through the analysis of melting curve profiles.

#### 3.2.4. Transferrin Receptor Quantification

Transferrin receptor levels were quantified using the Transferrin Elisa kit, respecting the manufacturer’s provided protocol. 10^5^ cells/well glioblastoma U87 cells were seeded onto the poly-L-lysine coverslip 12-well plate. After synchronization, cells were also collected every 4 h through a cycle of 24 h at T0, T4, T8, T12, T16, and T20 time points. The cell culture medium was taken out of the wells, and the cells were washed three times with PBS. Cell pellets were obtained after trypsin incubation for 3 min and centrifugation at 5000 rpm for 5 min. Cell lysis occurred by the addition of a buffer composed of 1% Triton X-100 detergent, 0.1% SDS in PBS, pH 7.4, and protease inhibitor cocktail. After cell recovery and lysis, samples were quantified and pipetted to a microtiter plate and receptor absorbance detected at 450 nm using a Shimadzu UV–vis 1700 spectrophotometer (Shimadzu, Duisburg, Germany). The mechanism consists of the linkage of transferrin receptor to a biotin-labelled capture antibody pre-linked to a streptavidin-coated microtiter plate. After that, a peroxidase-labelled detection antibody reacts with the substrate tetramethylbenzidine (TMB) compound to form a coloured product measurable spectrophotometrically.

#### 3.2.5. Formulation and Characterization of Complexes

The synthesis and preparation of TMZ-loaded peptides, as the formulation and characterization of TMZ/WRAP5/pDNA complexes, were already presented and discussed in previous publications from our research group [29,32]. TMZ drug loading efficiency (DLE) in the WRAP5 and Tf-WRAP5 peptides was calculated, resulting in 60.1% and 66.4% efficiency, respectively. Solutions of N/P (nitrogen to phosphate groups ratio) of 1 were prepared for 1 µg plasmid DNA (pDNA) encapsulation. 50 µL of this CPP ratio solution was pipetted into pDNA solution dropwise at vortex for 60 s. The solutions were kept at room temperature for 25 min for the complexes’ self-assembling, and then centrifuged at 13,500× *g* for 20 min at 4 °C. After centrifugation, the pellet containing the formed complexes was recovered. WRAP5/pDNA complexes were subsequently characterized by dynamic light scattering (DLS) regarding their mean size, zeta potential, and polydispersity index (PdI) and in terms of morphology. Details regarding these experiments are fully described elsewhere [29,32]. TMZ/WRAP5/pDNA complexes presented a mean size of 232.7 nm, a zeta potential of +4.43 mV, and a PdI of 0.327, while TMZ/Tf-WRAP5/pDNA complexes appeared with a mean size of 182.9 nm, a surface charge of +11.94 mV, and a PdI of 0.396. Additionally, using the Nano-Photometer™ device (Implen, Inc., Westlake Village, CA, USA) a pDNA complexation capacity (CC) of 94.33% and 89.56% was achieved for both complexes, respectively.

#### 3.2.6. Live Cell Imaging Assay

##### FITC Plasmid Labelling

The protocol for pDNA labelling with FITC started by mixing 81 µL of 0.1 M sodium tetraborate, pH 8.5, with 2 µg of pDNA and 2 µL of FITC (50 mg/100 µL of anhydrous dimethyl sulfoxide (DMSO)). Eppendorfs were protected from light and maintained in constant agitation in a rotating tube mixer for 4 h at room temperature. To stop the labelling reaction, 1 volume of 3 M NaCl and 2.5 volumes of 100% ethanol were added to the mixture, and samples were kept at −20 °C overnight. The next day, samples were centrifuged at 10,000× *g* for 30 min, at 4 °C, and all the ethanol was removed and replaced by ultrapure grade water for posterior formulation with TMZ-loaded CPP.

##### Cellular Uptake

To monitor cellular internalization, U87 cells were seeded at a density of 10^4^ cells/well onto the µ-slide 8-well (Ibidi, Martinsried, Germany) and grown in 250 µL of medium. Immediately before the live cell experiment, the nucleus was stained with a Hoechst 33342 solution (5 µg/mL, PBS) for 10 min, washed with PBS, and the cells were returned to the usual medium. Transfection mediated by the complexes (0.1 µg of pDNA-FITC) was performed at T0 and T8 time points, the time points with lower and higher expression of transferrin receptor, respectively. Through a live cell experiment using a Zeiss LSM 710 confocal laser fluorescence microscope (CLSM) (Carl Zeiss SMT, Inc., Oberkochen, Germany), complexes’ internalization was followed up during 1 h with ZEN microscopy software 3.7. During fluorescence images’ acquisition, Hoeschst 33342 (Diode 405-30 laser unit, λ = 405 nm) and Hoechst 33342 (Argon/2 laser unit, λ = 488 nm) lasers were used. To process obtained images the ZEISS ZEN 3.7 software was used. In the end, a Corrected Total Cell Fluorescence (CTCF) equation was applied to determine the level of cellular fluorescence in fluorescence microscopy images analyzed by ImageJ.

CTCF = Integrated density − (Area of interest × Mean fluorescence of background grey values) (1)

#### 3.2.7. TP53 Expression Evaluation

The p53 mRNA expression was quantified by RT-qPCR at T4, T8, T16, and T20 time points. U87 cells were seeded at a density of 10^5^ cells/well onto the poly-L-lysine coverslip 12-well plate and grown in 1.5 mL of medium. The transfection with 1 µg of pDNA occurred for 4 h, and subsequently, cells were returned to their usual culture medium and collected after 12 h. The RNA extraction, cDNA synthesis, and RT-qPCR experiments were carried out as described above. To detect amplicons, a protocol with a final annealing/extension step at 60 °C for 30 s was carried out.

#### 3.2.8. p53 Expression Evaluation

The expression levels of the p53 were quantified at time points T4, T8, T16, and T20 using the p53 pan ELISA kit (Roche Applied Science, Penzberg, Germany), according to the manufacturer’s instructions. U87 cells were seeded in 12-well plates (10^5^ cells per well). To enhance transfection efficiency, cells were serum-deprived for a minimum of 12 h prior to the transfection. On the day of transfection, 1 µg of pDNA was added to each well and incubated for 4 h. Transfection was terminated by replacing the medium with a fresh complete culture medium. Cells were harvested 24 h post-transfection. After centrifugation to collect the cell pellets, lysis was performed using the cell lysis buffer. The p53 levels were then measured spectrophotometrically at 450 nm using a Shimadzu UV–vis 1700 spectrophotometer (Shimadzu, Duisburg, Germany).

#### 3.2.9. Statistical Analysis

Analysis of variance (ANOVA) was considered in all GraphPad Prisma software V9.0.0 (GraphPad Software Inc., New York, NY, USA) statistical analysis, with the addition of the Bonferroni test and a confidence level of 95% (*p* ≤ 0.05). For statistical significance analysis: ns, *p* > 0.05; *, *p* ≤ 0.05; **, *p* ≤ 0.01; ***, *p* ≤ 0.001; ****, *p* ≤ 0.0001.

CircWave v1.4 analysis software (Dr. Roelof A. Hut, http://www.euclock.org, accessed on 28 December 2021) served for circadian oscillation pattern analysis, using the harmonic regression method (α = 0.05). *p*-values obtained after analysis resulted from the software applied F-test.

CosinorPy, a GitHub Python package (CosinorPy 2.1) [50], was used to fit a cosinor model to circadian rhythm data to detect rhythmicity and evaluate rhythmicity-related parameters [50,72]. The regression model is written below:

(2) $$Y\;(t)=M+A\cos\left(\frac{2\pi t}{\tau }+\text{Ø}\right)+e(t)$$

where M represents the MESOR (Midline Statistic Of Rhythm), A refers to the amplitude, Ø is the acrophase, τ is the period of one cycle, and e(t) is the error term [50]. F-test evaluated whether the single and multi-component cosinor models were statistically significant to the data and the goodness of fit (α = 0.05).

## 4. Conclusions

Glioblastoma is a highly malignant brain tumour characterized by resistance to conventional treatments. Despite the implementation of current therapeutic strategies, the prognosis remains poor. It is therefore essential to investigate novel and alternative therapeutic approaches to enhance treatment efficacy and address current limitations. Chronotherapy, which aligns therapeutics with the patients’ circadian rhythms, has shown potential for improving therapeutic outcomes in certain diseases by optimizing drug administration timing. Moreover, gene therapy and technology, particularly through the use of CPPs, present promising strategies for improving the co-delivery of therapeutic drugs and nucleic acids. These approaches enhance cellular uptake, increase treatment targeting, and minimize side effects, ultimately contributing to more effective and safer glioblastoma therapies. The integration of chronotherapy, nanotechnology, and gene therapy altogether may represent a multidisciplinary approach aimed at improving treatment delivery and efficacy while minimizing side effects. In line with this, our team previously designed and developed transferrin WRAP5/TMZ-based complexes targeted to glioma cells to deliver a plasmid coding for the tumour suppressor p53. Despite complexes exhibiting compatibility with different cell lines and a zebrafish model, target-directed capability and enhanced cellular penetration, p53 expression, with promotion of apoptosis, no association between glioma cell circadian rhythms and complexes uptake/delivery performance was considered. In the present work, for the first time, the study of the expression of core clock genes like *Bmal1* and *Per2* in U87 glioma cells was conducted, by CircWave and CosinorPy models, and demonstrated their circadian expression profiles for at least 48 h with specific time points representing higher and lower mRNA expression levels. Transferrin receptor quantification data also demonstrated that this receptor is rhythmically expressed and exhibited a peak of activity around T8. After that, a confocal microscopy experiment evidenced the higher ability of complexes to uptake at the T8 time point, resulting from a higher cell membrane surface transferrin receptor expression at this time point. A more efficient *TP53* delivery to U87 cells nucleus and higher p53 mRNA levels was obtained for the same time point. Lastly, p53 content evaluation supported the well-known temporal difference between mRNA expression and protein translation and stabilization, with p53 levels reaching its highest value at T16. These results point to T16 as a potentially effective time point for enhancing therapeutic outcomes in U87 glioblastoma cells.

Overall, our team reported the relevance and potential therapeutic asset of tuning the time to enhance the targeting and cellular uptake of TMZ/Tf-WRAP5/pDNA complexes and, therefore, achieve higher drug/gene delivery and, thus, therapeutic outcomes.

## Figures and Tables

**Table 1:** Significance (*p*-value) and centre of gravity (COG) for *Bmal1*, *Per2*, and transferrin determined by CircWave analysis.

Gene	*p*-Value	Centre of Gravity (COG) (h)
*Bmal1*	0.028	15.71
*Per2*	0.022	8.14
Transferrin	0.005	6.78

**Table 2:** Summary of rhythmicity parameters obtained with the single component cosinor model.

Gene	*p*-Value	Period (τ)	MESOR (M)	Amplitude (A)	Acrophase (Ø) (Radians)	Centre of Gravity (COG) (h)
24 h						
*Bmal1*	0.05	4.94	1.30	0.60	−1.50	18.82
*Per2*	0.03	15.36	1.00	0.50	3.01	7.36
Transferrin	0.09	22.31	1.05	0.45	−1.13	20.00
48 h						
*Bmal1*	0.002	21.86	1.25	0.65	2.22	7.72
*Per2*	0.001	29.42	1.35	0.85	−2.26	33.42

**Table 3:** Summary of rhythmicity parameters obtained with the CircWave and single component cosinor model.

Software	*p*-Value	Period (τ)	MESOR (M)	Amplitude (A)	Acrophase (Radians)	Centre of Gravity (COG) (h)
CircWave	0.000	24.00	-	-	-	9.59
CosinorPy	0.083	25.11	199.95	120.71	−2.00	16.00

## Data Availability

Data are contained within the article and Supplementary Materials.

## Supplementary Materials

The following supporting information can be downloaded at: https://www.mdpi.com/article/10.3390/ijms26136130/s1.
