# Supplementary material for: Circadian-Tuned Peptide Drug/Gene Co-Delivery Nanocomplexes to Enhance Glioblastoma Targeting and Transfection
Source: Int J Mol Sci. 2025 Jun 26;26(13):6130. doi: 10.3390/ijms26136130 (PMC12250532; doi:10.3390/ijms26136130)
Supplement: Supplementary file 1 [file ijms-26-06130-s001.zip › ijms-3662211-supplementary.pdf]

Supplementary Materials

Circadian-tuned peptide drug/gene co-delivery nanocomplexes to enhance glioblastoma targeting and transfection

Ana Raquel Neves, Eric Vivès, Prisca Boisguérin, Telma Quintela and Diana Costa

Methods

RNA Extraction

For RNA cell extraction purposes, culture media from the well plates were aspirated, and cells were washed three times with phosphate buffer solution (PBS). To collect and extract total RNA, TripleXtrator (GRiSP GB23.0100, Porto, Portugal) (300 μL) was added to each well and cells left at room temperature for 5 min, followed by the addition of 60 μL of chloroform and vigorous stirring with a vortex for 15 s. Samples were kept at room temperature for 15 min and centrifuged at 12,000 g for more 15 min. The aqueous phase containing the cells' RNA was then transferred to a new Eppendorf tube, mixed by up and down with 150 μL of isopropanol, and left for 15 min at room temperature. After a centrifugation at 12,000 g for 15 min, the RNA pellet was washed with 150 μL of 75% ethanol (diluted in diethyl pyrocarbonate (DEPC) treated water), centrifugated at 12,000 g for 5 min, left to dry to air and in the end resuspended in 20 μL of DEPC treated water. The obtained RNA samples were quantified and its quality analysed using a NanoPhotometer™.

Bmal1 and *Per2* gene as analysed by conventional PCR

To detect cDNA expression levels of core-clock genes, a Speedy NZYTaq 2x Green Master Mix kit (NZYtech, Lisbon, Portugal) was used. Samples were afterwards placed in a T100™ Thermal Cycler (Bio-Rad Laboratories, Inc, Hercules, California, USA) and run on a 1% agarose gel. Primer's sequence and reaction set-up conditions are listed in Table S1-2. Finally, samples were analysed by electrophoresis at 130 V for 40 min and gels visualized under UV light in a Uvitec Fire-Reader system (UVItec Limited, Cambridge, United Kingdom).

Table S1. Conventional PCR reaction set-up and cycling parameters for core-clock genes analysis.

Primers used were diluted 1:20 from stock in ultra-pure grade water. GAPDH was used for expression normalization.

Gene	Primer reverse	Primer forward	Product size (bp[1])
Bmal1	5’-TTC CCT CGG TCA CAT CCT AC-3’	5’-TGC CAC CAA TCC ATA CAC AG-3’	123
Per2	5’-TCC GCT TAT CAC TGG ACC TT-3’	5’-TTG GAC AGC GTC ATC AGG TA-3’	109
**TFRC[2]**	5’-CAG GCT GAA CCG GGT ATA TGA-3’	5’-AGG ACG CGC TAG TGT TCT-3’	111
TP53	5’-CTG AGT CAG GCC CTT CTG TCTT -3’	5’-GAG CTG AAT GAG GCC TTG GA-3’	151
GAPDH[3]	5’-ATG TGG GCC ATG AGG T-3’	5’-TCA TTT CCT GGT ATG ACA ACG-3’	64

[1]bp – base pair; [2]TFRC - Transferrin receptor; [3]GAPDH - Human Glyceraldehyde 3-phosphate dehydrogenase.

Table S2. Conventional PCR reaction set-up and cycling parameters for core-clock genes analysis.

**Primer reverse**	0.4 µM
**Primer forward**	0.4 µM
**Template cDNA**	1.25 µl
**Cycling parameters**<br>**(3-step protocol)**	Annealing: 60 °C<br>Cycles: 35

Results

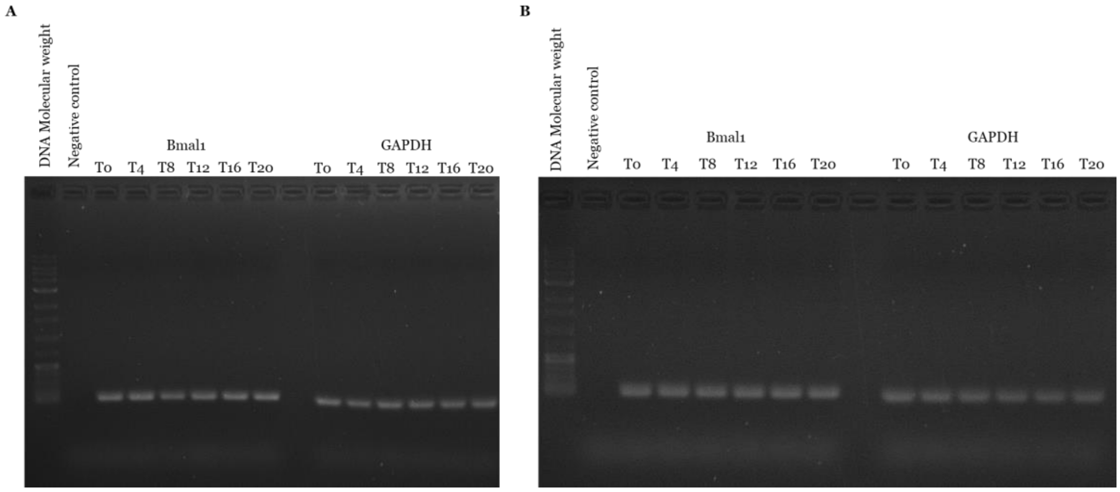

Figure S1. Electrophoretic analysis of the PCR amplicon band for *Bmal1* and *GAPDH* genes. **A** Electrophoretic analysis during 24 h. **B** Electrophoretic analysis during 48 h. Lane 1: DNA molecular weight marker; Lane 2: sample without cDNA; Lanes 3/4/5/6/7/8: gene expression at each time-point.

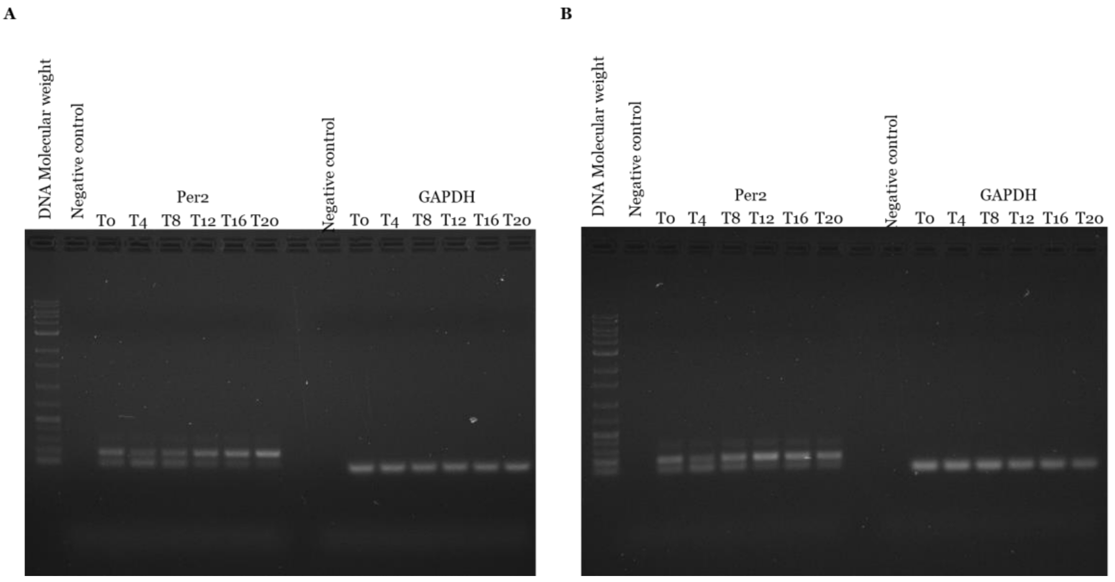

Figure S2. Electrophoretic analysis of the PCR amplicon band for *Per2* and *GAPDH* genes. **A** Electrophoretic analysis during 24 h. **B** Electrophoretic analysis during 48 h. Lane 1: DNA molecular weight marker; Lane 2: sample without cDNA; Lanes 3/4/5/6/7/8: gene expression at each time-point.

Table S3. Statistic parameters obtained for single and multi-component cosinor models.

**Gene**	**Residual Sum of Squares (RSS)**		**Mean Squared Error (MSE)**	
	Single	Multi	Single	Multi
**24 h**				
Bmal1	32554.419	32554.419	32.554	32.554
Per2	314730.244	314730.244	314.730	314.730
**Transferrin**	663980.290	663980.290	663.980	663.980
**48 h**				
Bmal1	637465.025	637465.025	637.465	637.465
Per2	1154626.135	1154626.135	1154.626	1154.626
